# Study protocol: realist evaluation of effectiveness and sustainability of a community health workers programme in improving maternal and child health in Nigeria

**DOI:** 10.1186/s13012-016-0443-1

**Published:** 2016-06-07

**Authors:** Tolib Mirzoev, Enyi Etiaba, Bassey Ebenso, Benjamin Uzochukwu, Ana Manzano, Obinna Onwujekwe, Reinhard Huss, Nkoli Ezumah, Joseph P. Hicks, James Newell, Timothy Ensor

**Affiliations:** 1 Nuffield Centre for International Health and Development, University of Leeds, Charles Thackrah Building, 101 Clarendon Road, Leeds, UK; 2 Health Policy Research Group & the Department of Health Administration and Management, College of Medicine, University of Nigeria Enugu-Campus, Enugu, 400001 Nigeria; 3 School of Sociology and Social Policy, Social Sciences Building, University of Leeds, Leeds, UK

**Keywords:** Realist evaluation, Community health workers, Maternal and child health, Conditional cash transfer, Nigeria, Mixed methods, Health systems research

## Abstract

**Background:**

Achievement of improved maternal and child health (MCH) outcomes continues to be an issue of international priority, particularly for sub-Saharan African countries such as Nigeria. Evidence suggests that the use of Community Health Workers (CHWs) can be effective in broadening access to, and coverage of, health services and improving MCH outcomes in such countries.

**Methods/design:**

In this paper, we report the methodology for a 5-year study which aims to evaluate the context, processes, outcomes and longer-term sustainability of a Nigerian CHW scheme. Evaluation of complex interventions requires a comprehensive understanding of intervention context, mechanisms and outcomes. The multidisciplinary and mixed-method realist approach will facilitate such evaluation. A favourable policy environment within which the study is conducted will ensure the successful uptake of results into policy and practice.

A realist evaluation provides an overall methodological framework for this multidisciplinary and mixed methods research, which will be undertaken in Anambra state. The study will draw upon health economics, social sciences and statistics. The study comprises three steps: (1) initial theory development; (2) theory validation and (3) theory refinement and development of lessons learned. Specific methods for data collection will include in-depth interviews and focus group discussions with purposefully identified key stakeholders (managers, service providers and service users), document reviews, analyses of quantitative data from the CHW programme and health information system, and a small-scale survey. The impact of the programme on key output and outcome indicators will be assessed through an interrupted time-series analysis (ITS) of monthly quantitative data from health information system and programme reports. Ethics approvals for this study were obtained from the University of Leeds and the University of Nigeria.

**Discussion:**

This study will provide a timely and important contribution to health systems strengthening specifically within Anambra state in southeast Nigeria but also more widely across Nigeria. This paper should be of interest to researchers who are interested in adapting and applying robust methodologies for assessing complex health system interventions. The paper will also be useful to policymakers and practitioners who are interested in commissioning and engaging in such complex evaluations to inform policies and practices.

**Electronic supplementary material:**

The online version of this article (doi:10.1186/s13012-016-0443-1) contains supplementary material, which is available to authorized users.

## Background

In this paper, we report a protocol for realist evaluation study of Dete*R*minants of *E*ffectiveness and sustainability of a no*V*el Community He*A*lth Workers (CHWs) program*M*e in im*P*roving maternal and child health in Nigeria (REVAMP project).

Achievement of improved maternal and child health (MCH) outcomes continues to be an issue of international priority for achieving the health-related Sustainable Development Goals (SDGs), particularly for sub-Saharan African countries [1]. In Nigeria, for example, despite the significant reduction in maternal and neonatal mortality since 2003 by 50 and 23 %, respectively, these still remain high at 545/100,000 and 37/1000 births, respectively, particularly in rural areas where most vulnerable groups reside [2, 3].

Evidence suggests that the use of CHWs can be effective in broadening access to, and coverage of, health services and improving MCH outcomes [4–6]. The use of CHWs was promoted in an attempt to implement interventions using lower cadres of workers to accelerate achievement of universal healthcare coverage [7–10].

However, to guide further development and implementation of CHW programmes in different contexts, it is necessary to better understand what makes CHW programmes successful in achieving desired outcomes and under what circumstances they succeed [11, 12]. As new programmes emerge or existing programmes scale up, comparing the effectiveness of CHW programmes between different contexts becomes important. This is due to the diversity of country contexts and complexity of CHW programmes, which require combinations of elements at micro (i.e. individual), meso (organisational) and macro (i.e. system) levels [7, 13]. Studies have explored the effectiveness of CHW programmes in improving MCH outcomes [14, 15], productivity of CHWs [12], costs and cost-effectiveness of CHW initiatives [4, 16], power relations and acceptance of CHW programmes by the public [6, 17] and effectiveness of associated conditional cash transfers (CCTs) linked to uptake of services [18, 19]. While studies have explored the effects of supply- and demand-side interventions separately [15, 18, 19], the combined effects of the two—such as the added value of CCTs within health programmes—are rarely assessed within the same intervention.

CHW programmes are inherently complex, and their success is mediated by how the intervention is implemented within the health system context. Theory-driven forms of evaluation help understanding such complexity by studying how the different elements are intertwined [20] and recognising the role of context as a key influence in the production of outcomes [21]. The UK Medical Research Council (MRC) guidance for evaluating complex interventions also states that process evaluation can help ‘assess fidelity and quality of *implementation*, clarify *causal mechanisms* and identify *contextual factors* associated with variation in outcomes’ [22].

Realist evaluation (RE) is a theory-driven evaluation method that is increasingly used for studying the implementation of complex interventions within health systems, including in low- and middle-income countries [23–25]. A realist approach emphasises the contingent nature of programme outcomes and addresses questions about what works, in which setting, for whom, in what circumstances and why [26]. In RE, researchers develop *middle-range theories* that take account of how *Context* (at micro, meso and macro levels) influences intervention processes or *Mechanisms* (e.g. actors’ behaviours in implementing intervention) to produce intended and unintended *Outcomes*. This is known as the C-M-O configuration [26], and without accounting for all these dimensions, some aspects of the programme may go unrecorded, thus affecting the validity and reliability of results [27] and preventing replication [28].

In 2012, the Federal Government of Nigeria launched the Subsidy Reinvestment and Empowerment Programme (SURE-P) to invest the revenue from fuel subsidy reduction into a social protection programme to improve the lives of the most vulnerable populations [29]. One SURE-P component focused on maternal and child health (SURE-P/MCH), which comprised supply and demand components. Central to the supply component was the recruitment, training and deployment of 2000 formal service providers (e.g. skilled midwives) and 10,000 CHWs, comprising 1000 Community Health Extension Workers (CHEWs) and 9000 volunteer Village Health Workers (VHWs). The supply component also included infrastructure development, improving availability of supplies and medicines and activation of Ward Development Committees (WDCs). This combination was intended to improve access to quality health services and ultimately reduce maternal and child morbidity and mortality. The demand component of the SURE-P/MCH programme aimed to increase utilisation of health services during pregnancy and at birth through the use of CCTs, i.e. financial incentives to pregnant women to register at a Primary Health Care (PHC) centre, receive health check-ups while pregnant, deliver at a health facility and take their baby for vaccinations. CCTs also target Traditional Birth Attendants (TBAs) to incentivise them to accompany pregnant women to PHC facilities. Since December 2012, CCTs have been implemented in selected SURE-P/MCH sites in 9 of the 36 states of Nigeria (SURE-P/MCH + CCT). Preliminary evidence indicates that paying CCTs to pregnant mothers is linked to increase antenatal care visits and facility deliveries [2]. While the average number of PHC facilities in each Nigerian state is typically between 1000 and 4000, the SURE-P/MCH was implemented in clusters of 12 to 21 health centres within selected states. Phase 2 of the SURE-P/MCH was launched in late-2013 and aimed for an incremental expansion to a further 12 to 21 facilities in the selected states.

Key officials in the Ministry of Health at Federal, State and Local Government levels expressed interest in assessing the performance of SURE-P/MCH and SURE-P/MCH + CCT (the ‘interventions’) and key contextual influences on the achievement of their objectives. Based on this interest, and following the competitive evaluation of research proposals from the Joint MRC/ESRC/DFID/Wellcome Trust health systems research initiative call 1, in June 2015, we initiated a 5-year research programme to assess the MCH component in Anambra state. In October 2015, 6 months after being elected, the new President of Nigeria announced his decision to reverse fuel subsidy reduction in order to catalyse the economic growth, effectively withdrawing government funding to SURE-P. However, the interest from Nigerian health officials in learning lessons for improving MCH outcomes remained high. We discussed the best course of action for our research with Nigerian health authorities and our funders. Options discussed included stopping or amending the research, as well as technical and political implications of each option from the different (policymakers’ and the funder’s) perspectives. The eventual decision was to continue with the study, using the original methodology, though with the addition of assessment of sustainability of achieved changes and effects of on-going lobbying and advocacy efforts on entrenching the MCH on the political agenda in Nigeria.

The purpose of this paper is to share the study protocol for realist evaluation of CHW programme in Nigeria. In the discussion, we also use to share some key initial methodological outputs which were produced since the start of the project [30]. This paper should be of interest to researchers who are adapting and applying robust methodologies for assessing complex health systems interventions and policymakers and practitioners who are interested in commissioning, and engaging in, similar evaluations.

### Study objectives and location

The aim of this study is to inform strengthening, scaling up and ensuring sustainability of CHW programmes. This will be achieved by investigating two intervention strategies (i.e. with and without CCTs) of a Nigerian CHW programme to understand what contextual factors promote equitable access to quality services and examining the conditions under which these changes can be sustained following withdrawal of funding.

The specific objectives of the study are to:

1. Develop an in-depth understanding of the *context* and the *process of implementation* of the interventions, including relationships between health workforce and infrastructure and supplies
2. Identify, assess and compare the intervention *outputs* (e.g. skills and practices of CHWs and effectiveness and efficiency of Primary Health Care facilities) and *outcomes* (e.g. equitable access to quality MCH services and attainment of MCH outcome targets) during and after withdrawal of targeted support to the programme
3. Develop an empirically based and theoretically grounded dynamic *model* of complex relations between the actors, context, implementation process, outputs and outcomes of the interventions during, and after withdrawal, of targeted support to the programme
4. Assess the role of different advocacy and lobbying efforts in entrenching MCH on the political agenda and strengthening the provision of MCH services, following the suspension of targeted support to SURE-P/MCH
5. Develop transferable best practices for *scalability* (expansion within a broadly similar context) and *generalisability* (expansion to different contexts) of the lessons learned

The study is guided by the research questions shown in Table 1, alongside the corresponding objectives.

**Table 1:** Study research questions

Specific Research Questions	Relevant objective
1. What are the supply and demand mechanisms, including costs, by which CHW programmes can improve equitable access to quality MCH care in Nigeria?	1–3
2. What are the relationships between the different components of successful CHW programmes?	
3. Which contextual factors determine whether intervention mechanisms lead to intended outputs, and subsequently outcomes, in Nigeria?	
4. Which contextual factors determine whether the programme outputs and outcomes are sustained following withdrawal of targeted support?	
5. In what ways do different advocacy and lobbying efforts influence the entrenching of MCH on the political agenda?	4
6. In what ways do the entrenchment of MCH on the political agenda influence the provision of MCH services in Anambra State?	
7. What lessons for scaling up and sustainability can we learn from the assessment of implementation of CHW programme, during and after targeted support?	3, 5
8. What wider lessons can other programmes, and other countries, learn from the implementation of CHW programmes during and after targeted support in Nigeria?	

This 5-year study is implemented in Anambra state, located in the southeast of Nigeria with a total of 4420 PHC facilities serving a population of 4.2 million. It was identified in consultation with the Federal and State Ministry of Health (MOH) and the SURE-P national team lead. We will select three study clusters, corresponding to Local Government Areas (LGAs): one with SURE-P/MCH, one with SURE-P/MCH + CCT and one with no intervention. A cluster is made up of four PHC facilities and one General Hospital (GH). The two interventions (i.e. SURE-P/MCH and SURE-P/MCH + CCT) will be assessed against each other and against the no implementation site. LGAs will be selected in the discussion with State MOH policymakers and programme managers based on high maternal mortality ratios and existence of effective and committed district health leadership.

## Methods/design

### Study conceptual framework

*Realist evaluation* provides an overall methodological approach for the project and will guide development, testing and refining of middle-range theories through the analysis of the relationships between the context (at macro, meso and micro levels), mechanisms and outcomes [31]. We will use *social science methods* to explore views of key actor groups. *Economic evaluation* will be used to identify the programme costs against the outcomes [32, 33], using an incremental approach to compare intervention costs with benefits, compared with standard practice [16, 34]. *Statistical analysis of quantitative data* from the health management information system (HMIS) and SURE-P monitoring and evaluation (M&E) system will enable us to determine the extent to which the interventions achieved improvements in MCH services.

Mixed methods study designs that are increasingly used to evaluate complex interventions [35] include the *exploratory* (qualitative methods followed by quantitative), *explanatory* (quantitative then qualitative), *embedded* (one dataset provides a supportive secondary role) and *convergent* (both datasets are complementary to each other) models [35, 36]. We shall deploy the convergent model to allow continuous integration and triangulation of quantitative and qualitative findings.

**Fig. 1:**
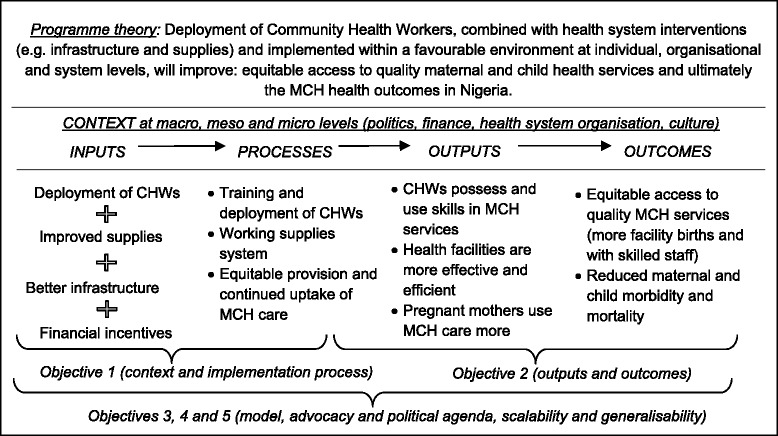
Conceptual framework for the study

The project uses an *input-process-output-outcome continuum* (see Fig. 1) to explore how inputs affect processes and how processes lead to outputs and ultimately outcomes. Implicit in the figure are the intervention implementation outcomes, i.e. acceptability, adoption, appropriateness, feasibility, fidelity, implementation cost, penetration and sustainability of the interventions [37], which often influence the progression from inputs to processes, outputs and outcomes within health programmes.

We will also assess the relationships within each stage: whether and how the different inputs complement each other; any catalysing or mutually negating effects between the processes (e.g. implications of staff absences due to training on availability and quality of MCH services) and any relations between the different outputs and between the different outcomes.

We recognise *context* as a key influence in achieving the intended results. Therefore, instead of *attributing* changes in health outcomes to the SURE-P/MCH only, we will explore the *contribution* of the interventions to achievement of desired effects within the real context. We will explore context at the macro level (e.g. political and resource environment), at the meso level (e.g. organisations and their roles) and micro level (e.g. capabilities, values and interests of individuals) [38, 39]. We will also assess the relationship between the supply and demand programme components, which are recognised to provide the continuum of care [40].

### Study design and methods

**Fig. 2:**
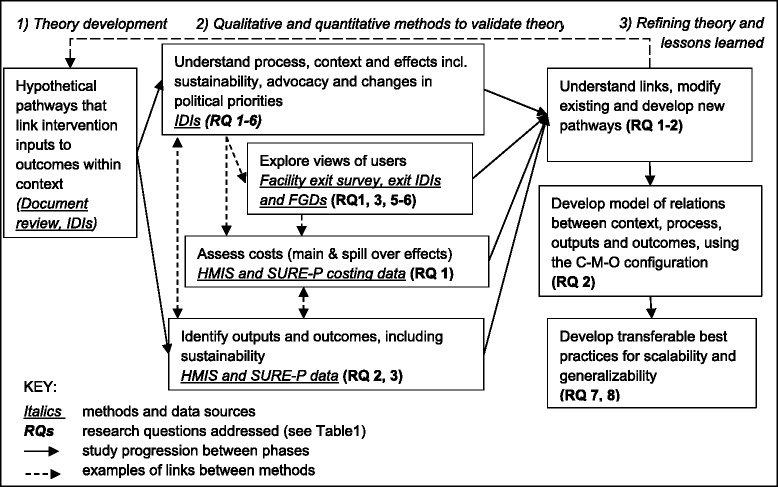
Study design and methods

The study methodology will include three steps (Fig. 2): (1) initial theory development; (2) theory validation and (3) theory refinement and development of lessons learned.

During *step 1*, we will develop specific hypothetical pathways (i.e. middle-range theories) that link intervention inputs to processes, outputs and outcomes within the context of Anambra state. The SURE-P programme theory (see Fig. 1) provides an overarching hypothesis, and more specific hypothetical pathways will represent the middle-range theories (MRT) to help us explore the C-M-O configurations within the programme. These hypotheses will be developed in discussions with programme managers and implementers.

The specific hypotheses will draw upon two data collection methods: (a) *review of key documents* (SURE-P implementation manual, relevant federal and state-level policies, e.g. reproductive and/or MCH, health workforce) and (b) a limited number (3–5 in total) of initial *in-depth interviews (IDIs)* with purposefully selected federal and state SURE-P programme managers.

In addition to assessing whether the target indicators are achieved, we will identify how they are achieved (i.e. exploring the intervention mechanisms); identify the key contextual facilitators and constraints and analyse in what ways these contextual influences affected the achievement of outcomes.

The specific hypotheses will be driven by the study research questions (see Table 1) and will relate to the key programme targets ([40] p. 17)

- Reduction of MMR by 59 % from 545/100,000 live births to 320/100,000 live births
- Reduction of neonatal mortality rate by 22 % from 37/1000 live births to 29/1000 live births
- Increased percentage of pregnant women receiving focused antenatal care (ANC) by 52% from 50 % coverage
- Increased percentage of skilled birth attendance by 63 % (from 16 % baseline)
- Increased postnatal care attendance within 2 days of birth by 63 % (from 16 % baseline)
- Increased family planning attendance by 26 % (from 1 % baseline)

The hypotheses can cover more than one indicator. An example involving the first two indicators might be: ‘deployment of CHWs, combined with improvement in infrastructure and supplies when implemented within the health systems context of Nigeria, will help reduce maternal and neonatal mortalities to 320/100,000 and 7/1000 live births respectively’.

The hypotheses can also cover links within and between components of the conceptual framework (Fig. 1), i.e. intra-component and inter-component, respectively. An example of the former is: ‘training and deployment of CHWs combined with a working supplies system, and implemented in the context Nigerian health system, will achieve increase in skilled birth attendance by over 60 % and improve equitable provision of MCH care’. An example of the latter is: ‘financial incentives, combined with increased access to MCH care following deployment of CHWs and improved infrastructure will improve the uptake of antenatal care by pregnant mothers (by over 50 %)’.

During *step 2*, we shall use a mix of methods to validate hypothetical pathways. Qualitative methods will include (a) IDIs with key actors, including service users, (b) reviews of key documents and (c) exit IDIs and focus group discussions (FGDs) with service users and their family members. Quantitative methods will include analysis of quantitative and costing data from (a) HMIS, (b) SURE-P M&E and (c) a structured facility exit survey. The qualitative and quantitative methods will be integrated throughout to answer the eight study research questions.

Using *IDIs*, we will explore actors’ understandings and views about the intervention’s context and processes (or mechanisms), their expected and unexpected effects (outputs and outcomes) and effects of advocacy and lobbying in entrenching the MCH on the political agenda. This will give us greater understanding of the health system context, including the links with relevant policies, practices and programmes (e.g. consistency of SURE-P management with overall governance approaches; or support to CHWs within staff supervision and performance appraisal systems).

The respondents for IDIs, identified through purposive sampling, will include health facility managers, CHWs, PHC staff and health planners and programme managers at local, state and federal levels. A detailed list of respondents will be developed within step 1, and snowballing will be used to identify any further informants. We expect to have 30–45 IDIs to represent views of key actor groups in Anambra state (10–15 per each cluster) and about 30 at federal level. However, if we reach data saturation earlier, these numbers may decrease.

The development of interview question guides will be informed by the study conceptual framework and structured around the study research questions to explore the specific hypothetical pathways identified in step 1. Question guides will be adapted to the different actor groups, commensurate to their backgrounds and roles in the design and implementation of the programme. All interviews will be audio-recorded (subject to informed consent), transcribed and translated into English where required. A framework approach will be used for analysis to test hypotheses, while allowing for emergence of new themes [41].

The impact of the programme on a range of key output and outcome indicators will be assessed through an interrupted time-series analysis (ITS) of monthly (the unit of analysis) *quantitative data from HMIS and SURE-P M&E monthly programme reports*. The aim of the ITS analysis is to identify discontinuities in the time series associated with, and potentially caused by, the introduction of the SURE-P programme to health facilities. These include both immediate changes in the level of an indicator following the intervention, and long-term changes in the trend of an indicator (over time) following the intervention, as compared to control health facilities.

A general model for an ITS regression analysis (which will be adapted to allow for comparison between SURE-P/MCH, SURE-P/MCH + CCT and control clusters) is:

$$ {Y}_t = {\beta}_0+\kern0.5em {\beta}_1{T}_t+\kern0.5em {\beta}_2{I}_t+\kern0.5em {\beta}_3{T}_t{I}_t + {\beta}_4{X}_t + {\varepsilon}_t $$

where *Y*_*t*_ is a dependent outputs (e.g. delivery by skilled birth attendants, facility based delivery, ANC 4+, postnatal care within 48 h) or outcome (e.g. neonatal and perinatal deaths); *T*_*t*_ is a time series trend variable; *I*_*t*_ is an intervention dummy variable (taking a value of 1 in areas where the intervention is implemented and 0 otherwise); *T*_*t*_ *I*_*t*_ is time after the intervention; *X*_*t*_ is a covariate and *ε*_*it*_ is an error term. The parameter *β*_0_ represents the baseline level of the dependent variable; *β*_1_ represents the baseline trend in the dependent variable; *β*_2_ isolates any shift in the level (intercept) of the dependent variable following the introduction of the policy; *β*_3_ isolates any shift in the rate of change (slope) following the introduction of the policy and *β*_4_ represents the effect of a covariate.

The datasets will consist of indicator panels comprising health facilities within the three clusters in Anambra state where the two variations of the intervention are occurring (SURE-P/MCH and SURE-P/MCH + CCT, respectively), as well as a cluster where neither variation is implemented, which will function as a control. The panels will use data from each facility in the three study clusters covering the period from at least 12 months before and 12 months after the intervention began in the two intervention clusters and equivalent periods for the control cluster.

The quality of government and programme indicators will first be assessed by comparing the data with relevant indicators from the demographic surveillance system, and we will adjust the HMIS/M&E variables accordingly in the case of major discrepancies.

Findings from step 1 will be used to hypothesise the likely changes (interruptions in trends and levels) in the time series to make predictions to be verified during the ITS analyses. Methods will be used that adjust, where necessary, for problems typical to time-series estimation (e.g. autocorrelation), and important covariates will also be adjusted for, while multilevel methods will be used to address variation across health facilities. The assessment of the ITS design against standard quality checklist [42] is shown in Additional file 1.

This quantitative analysis interrelates with the qualitative IDIs mentioned earlier. For example, at the output level, we might expect that facility delivery rates will increase: the quantitative analysis will quantify the rate of change; the reasons for the change at this rate will then be explored using qualitative methods. At the outcome level, increased rates of facility delivery could help improve delivery outcomes, leading to a reduction in perinatal deaths. Conversely, it could also lead to the admission of more high-risk pregnancies, causing an increase in facility deaths.

In addition to clinical service outcomes shown in Fig. 1, the mixed methods approach will enable us to also analyse the intervention implementation outcomes, e.g. acceptability of the intervention by the communities and front-line service providers, appropriateness of the intervention design to the current context of PHC facilities in Anambra state and sustainability of changes achieved from the implementation of interventions in the longer term [37].

We will explore views of MCH service users on SURE-P, its costs and effects. The respondents will be current and former service users, recruited at the point of exiting from health facilities. The data will be collected using three methods. First, *small-scale facility exit survey* (about 300 respondents) will use structured questionnaire to explore user perceptions about the programme and their experiences in accessing MCH care. Second, *facility exit IDIs* will be conducted with 20–25 purposefully identified users to explore in-depth their views. Last, at least two *FGDs* will be conducted, each involving 6–8 service users including members of their families, to enable comparison of their views and exploring the dynamics of their discussion.

These service user views, together with qualitative IDIs, will inform the economic evaluation to understand the costs and cost-effectiveness of the programme and the contextual influences on the drivers of costs and benefits. This economic evaluation will use data from *analysis of costing data from HMIS and SURE-P* and will draw upon the results of the *facility exit survey*. Rather than attempting to cost all services, we will use an incremental approach that examines the additional programme benefits relative to the additional SURE-P costs. An incremental cost-effectiveness analysis will be undertaken (cost per additional skilled delivery and associated care).

We realise, however, that implementation of a new programme can have an impact that goes beyond the direct programme beneficiaries and will consider these effects qualitatively through conducting IDIs with key actors referred to earlier. The additional costs on the supply side are likely to be training midwives and CHWs, salaries, equipment and other supplies (Table 2). On the demand side, there are costs of the cash transfers themselves (conditional on using services), transportation to the health facility and opportunity costs of facility use to the women and their carers. From a wider social perspective, the impact of the interventions is likely to affect the costs of obtaining services. More accessible midwives/CHWs, for example, may reduce the cost to households of obtaining care.

**Table 2:** Key variables and considerations for economic evaluation

	Costs	Benefits	Action
Direct incremental	Direct programme	Change in	Use information to undertake an incremental cost-effectiveness analysis
	• Training, salaries, equipment	• Skilled deliveries	
	• Cash transfers incl. admin	• ANC 4 visits	
	Change in cost to users	• PNC attendances arising from the intervention	
	• Transport, opportunity cost		
Spill-over effects	Change in costs to other service providers	Change in	Use information to modify costs where possible; inform sensitivity analysis
		• General facility activity	
		• Service quality due to crowding, resource reallocation	

These costs will be captured through a small facility exit survey of women who have used services both before and after implementation of the SURE-P/MCH to understand how user costs have changed over time. These costs will be apportioned between the key SURE-P indicators: change in antenatal attendances, skilled deliveries and postnatal attendances.

Spill-over effects and unintended consequences of the programme will be tracked, quantified and also explored qualitatively. Evidence from elsewhere [43] is that demand-side programmes can impact on other services, through over-crowding and excessive bed-occupancy. Other services might also be affected by resources (or their lack) such as drugs and theatre time that are channelled into the new programme. These costs represent opportunity costs of the programme that contribute to the overall cost that may vary across contexts. We will use the understanding of the context in which interventions are operating to identify these consequences. We will also track significant non-quantifiable effects and will include estimates in the incremental cost-effectiveness analysis.

The uncertainty and likely variability of costs and effectiveness, particularly the spill-over effects, mean that sensitivity analysis will form an important part of the reporting of the economic evaluation. Sensitivity analysis captures the influence on cost-effectiveness ratios of change in major assumptions [16]. In addition to changes in exogenous variables such as pay scales and size of the programme, we will include key contextual changes that can impact on the cost-effectiveness of the programme. This may include the range of other services offered and occupancy levels.

During *step 3*, we will refine our hypothetical pathways, develop a model of the complex relations between the actors, context, intervention processes, outputs and outcomes, and develop transferable best practices for scalability and generalisability of the programme. This will allow us to answer most of our research questions. Although we do not anticipate any further data collection for this stage, we do not see a linear progression between the steps: i.e. as part of the analysis, we are likely to identify further pathways which may require further data collection and analysis.

The project workplan showing overlaps between the three steps is included in Additional file 2. The 5-year duration is feasible and should allow sufficient time for developing robust multidisciplinary methodology, assessing longer-term outcomes, conducting individual and organisational capacity strengthening [44], adequate consultations with actors and facilitating uptake of project results into policy and practice [45].

### Ethics and research governance

Ethical approvals for this study were obtained from the University of Leeds (ref: SoMREC/14/097) and the University of Nigeria (ref: NHREC/05/02/2008B-FWA00002458-1RB00002323). These are available in Additional files 3 and 4, respectively.

The project will be carried out with full respect of current relevant legislation (e.g. the Charter of Fundamental Rights of the EU) and international conventions (e.g. Helsinki Declaration). The methods development, data collection and analysis will take account of the following issues:

- *Anonymity* of study respondents will be preserved and ensured at all times as respondent(s) request. Unnecessary collection of personal data will be avoided, and respondents will have the right to review outputs and withdraw consent. All personal data will be coded, removed from the data for analysis and stored separately. Only designated research staff will have access to the keys linking the data with the personal information.
- *Informed consent* will be obtained from all study participants, and in the case of refusal, alternative means of data collection will be explored (e.g. alternative respondents)
- Specific emphasis will be placed on *confidentiality and other data protection issues*, which will include security of data storage and access rights to data. Only members of teams identified by the PIs in each institution will have access to the data. Where project data are stored on an institutional server, it will be password-protected and only members of the research team will have access to the passwords.

The project will be implemented according to standard governance practices at the University of Leeds and University of Nigeria. This includes ensuring regular communication between the partners and engagement with policymakers and practitioners; quality assurance through regular peer-review within and between the teams; appropriate mentoring and coaching support to more junior researchers and equal opportunities to both genders.

### Communication and dissemination of results

Adequate communication of results is an essential component of this study. We will ‘embed’ the research into policy and practice, working with the federal, state and local actors. This approach, developed by the Nuffield Centre, has been effective in many countries in improving the quality and effectiveness of the scaled-up programme [46]. The Health Policy Research Group, University of Nigeria has developed three models for getting research into policy and practice (GRIPP), which will also be applied in this study.

Decision-makers will be continuously engaged in a research-policy partnership to facilitate adoption of lessons learned [45]. Specific methods of communicating research will include combinations of:

- Developing short and practical policy briefs to national and international policymakers and practitioners
- Delivering presentations at local, state and federal meetings in Nigeria and relevant international meetings;
- Regular project review meetings and continuous engagement with key decision-makers
- Developing newsletters, press-releases and interviews in the media aimed at communicating the key project findings to the public in Nigeria and more widely
- Developing a dedicated website where the project results will be publicly accessible by national and international decision-makers, practitioners and academics
- Delivering presentations at national and international conferences and publication of articles in peer-reviewed academic journals with emphasis on open access
- Developing a project research report for the funder, with a publishable executive summary

## Discussion

Our study should improve understanding of the performance and functioning of complex system interventions involving both supply and demand sides. The study results will also inform strengthening the different aspects of the Nigerian health system, e.g. assessment of context will inform best practices in PHC staff performance management; assessment of the added value of CCTs will inform further demand-side financing schemes.

Since the start of the project, a detailed methodology handbook has been developed to guide data collection and analysis [30]. This handbook is available upon request. Two supplementary materials are included from this handbook. First, the initial Logic Model (LM) (see Additional file 5) was developed for the SURE-P/MCH. A LM is a visual way of organising and displaying information about a strategy or programme. A coherent LM is a thread of evidence-based logic that connects design, planning, implementation and evaluation of programmes [47]. LMs can assist stakeholders to untangle, clarify and share their understanding of complex relationships amongst programme elements [47]. The LM for SURE-P/MCH was developed using a combination of documents review, informal consultations with SURE-P/MCH manager and a technical workshop that involved researchers from the Universities of Leeds and Nigeria.

Second, two initial hypothetical pathways or initial working theories (IWTs) (see Additional file 6) were developed, focusing on SURE-P/MCH supply and demand components, respectively. These progressed from (1) overall programme theory, (2) initial LM and (3) literature review. Each IWT identified specific Cs, Ms and Os. The relationships between and amongst these Cs, Ms and Os will be explored as part of the data collection and analysis. The IWTs subsequently guided the identification of the specific information areas for the data collection and analysis.

Two aspects of the environment within which the study is being implemented are worth noting. First, the policy environment in Nigeria within which the research is being undertaken is favourable to ensure a high-quality analysis, inform theoretical debate and ensure the uptake of results into policy and practice, as we found within our previous collaborative projects. The commitment by key health decision-makers at Anambra state to engage with this research is particularly encouraging. Second, gaps in the literature on the CHWs, combined with an increasing interest in applied research from policymakers and funders, create a favourable environment for the study provide a timely contribution to an on-going debate about effectiveness of complex CHW interventions.

This study will make an important and timely contribution to health systems strengthening in Nigeria. Evaluation of complex interventions such as SURE-P and their longer-term impact on MCH outcomes requires a comprehensive understanding of intervention context, implementation, mechanisms and outcomes. The multidisciplinary and mixed methods realist approach that will be used in the study will facilitate such evaluation.

## Abbreviations

ANC, antenatal care; CCT, conditional cash transfers; CHEWs, Community Health Extension Workers; CHWs, Community Health Workers; FGD, focus group discussion; HMIS, health management information system; IDI, in-depth interview; IWT, initial working theory; M&E, monitoring and evaluation; MCH, maternal and child health; MOH, Ministry of Health; MRT, middle-range theory; PHC, Primary Health Care; REVAMP, dete*R*minants of *E*ffectiveness and sustainability of a no*V*el Community He*A*lth Workers program*M*e in im*P*roving maternal and child health in Nigeria; SURE-P, Subsidy Reinvestment and Empowerment Programme; VHWs, Village Health Workers; WDCs, Ward Development Committees.

## Additional files

Additional file 1: Assessment of ITS design against quality criteria. (DOCX 29 kb)

Additional file 2: Project workplan. (DOCX 22 kb)

Additional file 3: Ethics approval from the University of Leeds. (PDF 555 kb)

Additional file 4: Ethical approvals from the University of Nigeria. (ZIP 1568 kb)

Additional file 5: Pre-implementation Logic Map for SURE-P/MCH. (PDF 110 kb)

Additional file 6: Initial Working Theories. (DOCX 37 kb)
