# Supplementary material for: Study protocol: realist evaluation of effectiveness and sustainability of a community health workers programme in improving maternal and child health in Nigeria
Source: Implement Sci. 2016 Jun 7;11:83. doi: 10.1186/s13012-016-0443-1 (PMC4896007; doi:10.1186/s13012-016-0443-1)
Supplement: Supplementary file 1 — Assessment of ITS design against quality criteria. (DOCX 29 kb) [file 13012_2016_443_MOESM1_ESM.docx]

**Additional file 1: Assessment of ITS design against quality criteria**

| **Criterion [1]** | **Assessment of ITS design** |
| --- | --- |
| 1. Intervention occurred independently of other changes over time | The temporal effect of the SURE-P intervention on the outcomes being evaluated is unlikely to be fully independent, as there a number of possible (often complex) factors that may influence the outcomes in a time varying manner. For example, the Nigerian Midwives Service Scheme, which aims to increase midwife numbers in rural PHCs, and various health policies developed by the Nigerian MoH, and typically implemented through national programmes. However, the ITS design will compare outcomes in PHCs that have implemented SURE-P/MCH or SURE-P/MCH/+CCT to control PHCs where SURE-P was not implemented. Therefore, this will enable us to control for secular influences. In addition, part of the qualitative component of the project will assess contextual background influences, and their temporal changes, through the life of the project, which will also help in the interpretation of the ITS results in relation to understanding the effects of non-SURE-P influences. |
| 1. Intervention was unlikely to affect data collection | The data collection is based on secondary collection of routine hospital management information system data and additional data collected specifically in relation to the intervention, but not as part of this study. Therefore the intervention does not affect data collection. |
| 1. The primary outcome was assessed blindly or measured objectively 2. The primary outcome was reliable or was measured objectively | All data are based on formal records made by hospital staff who will not be blind to the format of the intervention or control present in their health facility. However, all outcomes are measured objectively, because they are all based on discrete, non-subjective events (e.g. child birth) or objectively measured continuous values (e.g. total staff salary). |
| 1. The composition of the data set at each time point covered at least 80% of the total number of participants in the study | Aside from any missing data, which is expected to be generally minimal given the use of routinely collected records, at each time point outcomes should cover 100% of the total number of participants in the study at that time point (i.e. 100% of patients in PHCs for whom the outcome is relevant, e.g. women attending ANC appointments). |
| 1. The shape of the intervention effect was prespecified | A statistical analysis plan will be produced prior to the ITS analysis which will pre-specify the shape of the intervention effect depending on the outcomes that are chosen to be analysed, but broadly it is expected that the implementation of the SURE-P MCH intervention will increase uptake of MCH services by pregnant women and the provision of MCH services by PHCs both immediately post-implementation, and increasingly over time as more women become aware of the services offered and their utilisation by their peers. Similarly, implementation of CCTs is expected to increase utilisation of MCH services by pregnant women both immediately post-implementation, and increasingly over time as more women become aware of the services offered and their utilisation by their peers. |
| 1. A rationale for the number and spacing of data points was described | At a minimum we will aim to have 12 data points for each intervention period, with the data being collected on a monthly basis, and therefore covering time periods that should be sufficient to observe any real changes in outcomes due to SURE-P’s effects on MCH service provision and utilisation at PHCs. Assuming an autocorrelation of 0.5 between time series data having a lag of 1, then 24 data points should provide 87% power to detect an effect size of 1 (defined as the expected intervention effect over its standard deviation, and assuming 20% of the change comes from a change in level and 80% from a change in trend) at a significance level of 0.05 [2]. |
| 1. The study was analysed appropriately using time series techniques | Prior to the ITS analyses a statistical analysis plan will be produced to determine which specific outcomes will be analysed, which hypothesis will be evaluated and what appropriate ITS techniques will be used to account for the complexities likely to be present in the time series data, such as temporal autocorrelation, clustering and heteroscedasticity. [3, 4]. |

**References**

1. Ramsay CR, Matowe L, Grilli R, Grimshaw JM, Thomas RE. Interrupted time series designs in health technology assessment: Lessons from two systematic reviews of behavior change strategies. International Journal of Technology Assessment in Health Care. 2003;19(4):613-23. PubMed PMID: WOS:000220479700003.

2. Zhang F, Wagner AK, Ross-Degnan D. Simulation-based power calculation for designing interrupted time series analyses of health policy interventions. Journal of Clinical Epidemiology. 2011;64(11):1252-61. doi: 10.1016/j.jclinepi.2011.02.007. PubMed PMID: WOS:000295605000012.

3. Linden A. Conducting interrupted time-series analysis for single- and multiple-group comparisons. Stata Journal. 2015;15(2):480-500. PubMed PMID: WOS:000357139500008.

4. Wagner AK, Soumerai SB, Zhang F, Ross-Degnan D. Segmented regression analysis of interrupted time series studies in medication use research. Journal of Clinical Pharmacy and Therapeutics. 2002;27(4):299-309. doi: 10.1046/j.1365-2710.2002.00430.x. PubMed PMID: WOS:000177464200009.
