# Supplementary material for: Study protocol: realist evaluation of effectiveness and sustainability of a community health workers programme in improving maternal and child health in Nigeria
Source: Implement Sci. 2016 Jun 7;11:83. doi: 10.1186/s13012-016-0443-1 (PMC4896007; doi:10.1186/s13012-016-0443-1)
Supplement: Supplementary file 2 — Project workplan. (DOCX 22 kb) [file 13012_2016_443_MOESM2_ESM.docx]

**Additional file 2: Project work plan**

| **Activity** | **Y1** | **Y2** | **Y3** | **Y4** | **Y5** |
| --- | --- | --- | --- | --- | --- |
| Development of detailed methodology for the study including tools for the data collection, piloting and post-piloting revisions as appropriate | X |  |  |  |  |
| Capacity building on use of realist evaluation and mixed methods research | X |  |  |  |  |
| Initial data collection and developing initial pathways(Step 1 in Figure 2) | X | X |  |  |  |
| Validating pathways using IDIs and developing baseline variables for project | X | X |  |  |  |
| Assessment of context, processes, outputs and outcomes of interventions, including sustainability of achieved changes (Step 2), including:   - Actors’ understanding and views about the programme and effects - Programme outputs and outcomes, including their sustainability - Views of users about the interventions and sustainability - Programme costs and cost effectiveness - Effects of lobbying and advocacy on entrenching MCH on the agenda |  | X | X | X |  |
| Comparative analysis of findings from the three clusters, developing model and transferable best practices (Step 3) |  |  | X | X | X |
| Repeated engagement with national and international actors, to communicate results and discuss policy and practice implications |  | X | X | X | X |
