# Supplementary material for: Study protocol: realist evaluation of effectiveness and sustainability of a community health workers programme in improving maternal and child health in Nigeria
Source: Implement Sci. 2016 Jun 7;11:83. doi: 10.1186/s13012-016-0443-1 (PMC4896007; doi:10.1186/s13012-016-0443-1)
Supplement: Supplementary file 3 — Ethics approval from the University of Leeds. (PDF 555 kb) [file 13012_2016_443_MOESM3_ESM.pdf]

# UNIVERSITY OF LEEDS

**Faculty of Medicine and Health Research Office**
**School of Medicine Research Ethics Committee (SoMREC)**

Room 10.111b, level 10
Worsley Building
Clarendon Way
Leeds, LS2 9NL
United Kingdom

✆ +44 (0) 113 343 1642

06 November 2015

Dr Tolib Mirzoev
**c/o Dr Bassey Ebenso**
Research Fellow
Nuffield Centre for International Health and Development
Leeds Institute for Health Sciences
University of Leeds
Room G.24, Charles Thackrah Building
101 Clarendon Road,
LEEDS LS2 9LJ

Dear Bassey

Ref no: **SoMREC/14/097**

Title: **Determinants of effectiveness of a novel community health workers programme in improving maternal and child health in Nigeria**

Your research application has been reviewed by the School of Medicine Ethics Committee (SoMREC) and we can confirm that local ethics approval is granted based on the following documentation received from you and subject to the following condition:

- **Evidence of ongoing ethical clearance from the University of Nigeria Teaching Hospital is submitted to this committee on expiry of the current certificate for the duration of the study**

| *Document* | *Version* | *Date Submitted* |
|---|---|---|
| REVAMP ETHICS APPLICATION FORM 1_SoMREC 14097_Vs 2 | 2 | 12/10/2015 |
| Annex 1: Ethical Clearance Certificate from the University of Nigeria Teaching Hospital, Enugu, Nigeria. | 1 | 20/07/2015 |
| Annex 2: Project proposal submitted to the Medical Research Council | | 20/07/2015 |
| Annex 3:<br>(i) Participant Information Sheet – Health Workers and Policy Makers<br>(ii) Participant Information Sheet – Questionnaire Survey for Service Users<br>(iii) Participant Information Sheet – Facility Exit Interviews for Service Users<br>(iv) Participant Information Sheet – Focus Group Discussions for Service Users<br>(v) and Family Members | 4 | 04/11/2015 |
| Annex 4: Project Data Management Plan | 1 | 20/07/2015 |

Please notify the committee if you intend to make any amendments to the original research ethics application or documentation. All changes must receive ethics approval prior to implementation. Please contact the Faculty Research Ethics Administrator for further information (FMHUniethics@leeds.ac.uk)

Ethics approval does not infer you have the right of access to any member of staff or student or documents and the premises of the University of Leeds. Nor does it imply any right of access to the premises of any other organisation,

including clinical areas. The committee takes no responsibility for you gaining access to staff, students and/or premises prior to, during or following your research activities.

*Please note:* You are expected to keep a record of all your approved documentation, as well as documents such as sample consent forms, and other documents relating to the study. This should be kept in your study file, which should be readily available for audit purposes. You will be given a two week notice period if your project is to be audited.

It is our policy to remind everyone that it is your responsibility to comply with Health and Safety, Data Protection and any other legal and/or professional guidelines there may be.

We wish you every success with the project.

Yours sincerely

**Dr Roger Parlsow**
**Co-Chair, SoMREC, University of Leeds**

**Dr Ruth Brooke**
**Co-Chair, SoMREC, University of Leeds**

*(Approval granted by co-Chair Dr Ruth Brooke, on behalf of School of Medicine Research Ethics Committee, Faculty of Medicine and Health, University of Leeds)*
