# Supplementary material for: Study protocol: realist evaluation of effectiveness and sustainability of a community health workers programme in improving maternal and child health in Nigeria
Source: Implement Sci. 2016 Jun 7;11:83. doi: 10.1186/s13012-016-0443-1 (PMC4896007; doi:10.1186/s13012-016-0443-1)
Supplement: Supplementary file 4 — Ethical approvals from the University of Nigeria. (ZIP 1568 kb) [file 13012_2016_443_MOESM4_ESM.zip › REVAMP_ProtocolPaper_AddFile_4_EthicsCOMUNECR1.pdf]

UNIVERSITY OF NIGERIA TEACHING HOSPITAL

ITUKU - OZALLA, P. M. B. 01129, ENUGU

TEL: 024 - 252022, 252573, 252172, 2552134, FAX: 042 - 252665
E-mail: cdunth@infoweb.abs.net
cmdunth2011@yahoo.com

Chief Sir Dr. C. J. UDEOGU, FICS
Specialist Surgeon, Endoscopist
Chairman UNTH Management Board

Barr. S. IKE NKUME,
LL.B(Hons); BL; MPA; B.Ed(Pol.Sc.);AHAN
Ag. Director of Administration/Secretary
UNTH Management Board

Dr. C. C. AMAH, MBBS, FWACS, FICS, FIAM, FNIM, FCE
Chief Medical Director

Dr. (MRS) ANNE C. NDU, MBBS, FWACP, MPH
Chairman Medical Advisory Committee

Our Ref: UNTH/CSA/329/Vol.5

Date: 6th March, 2015.

NHREC/05/01/2008B-FWA00002458-1RB00002323

ETHICAL CLEARANCE CERTIFICATE

TOPIC: **DETERMINANTS OF EFFECTIVENESS OF A NOVEL COMMUNITY HEALTH WORKERS PROGRAMME IN IMPROVING MATERNAL AND CHILD HEALTH IN NIGERIA.**

BY: ***PROF. OBINNA ONWUJEKWE***

FOR: **RESEARCH PURPOSE**

This research project on the above topic was reviewed and approved by the University of Nigeria Teaching Hospital Health Research Ethics Committee. This certificate is valid for **one year** from date of issue.

Prof. R.E. Umeh
Chairman, Health Research Ethics Committee

Date: 12/03/15

UNIVERSITY OF NIGERIA TEACHING HOSPITAL

ITUKU-OZALLA, P.M.B. 01129, ENUGU

E-mail: cmdunth2011@yahoo.com

Chairman UNTH Management Board

Barr. S. IKE NKUME,
LLB (Hons), BL, MPA; B.Ed(Pol. Sc.); AHAN
Director of Administration/Secretary
UNTH Management Board
08063388624
E-mail: ikenkume@gmail.com

Dr. C. C. AMAH, MBBS, FWACS, FICS, FNIM, FCE
Chief Medical Director
08033216393

Dr. OBINNA D. ONODUGO, MBBS, FWACP
Chairman Medical Advisory Committee
08033424217

Our Ref UNTH/CSA/329/OL.5

Date 12th April, 2016.

NHREC/05/01/2008B-FWA00002458-1RB00002323

ETHICAL CLEARANCE CERTIFICATE

TOPIC: DETERMINANTS OF EFFECTIVENESS OF A NOVEL COMMUNITY HEALTH WORKERS PROGRAMME IN IMPROVING MATERNAL AND CHILD HEALTH IN NIGERIA. ***(Renewed For 2016)***

BY: ***PROF. OBINNA ONWUJEKWE.***

FOR RESEARCH PURPOSE.

This research project on the above topic was reviewed and approved by the University of Nigeria Teaching Hospital Health Research Ethics Committee. This certificate is valid for **one year** from date of issue. Please note that the Committee Reserves the Right to monitor the Conduct of the study at any time for strict Compliance to the Protocol.

Prof. R.E. Umeh
Chairman, Health Research Ethics Committee

Date: 15/04/16
