# Supplementary figures and images for: Study protocol: realist evaluation of effectiveness and sustainability of a community health workers programme in improving maternal and child health in Nigeria
Source: Implement Sci. 2016 Jun 7;11:83. doi: 10.1186/s13012-016-0443-1 (PMC4896007; doi:10.1186/s13012-016-0443-1)

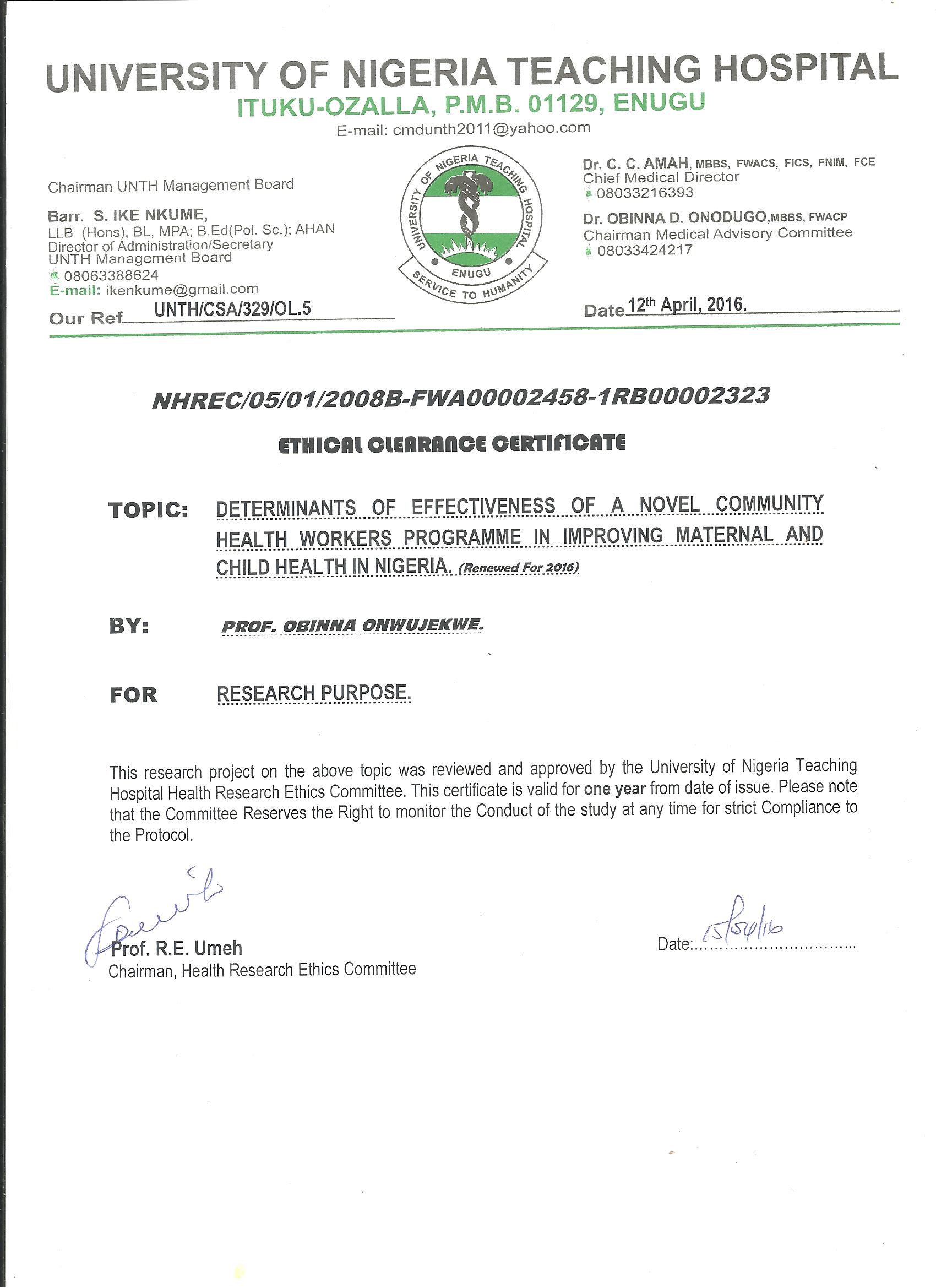

Supplement: Supplementary file 4 — Ethical approvals from the University of Nigeria. (ZIP 1568 kb) [file 13012_2016_443_MOESM4_ESM.zip › REVAMP_ProtocolPaper_AddFile_4_Ethics Approval_nigeria_renewedR1.jpg]

Additional file 5: Pre-implementation Logic Map for SURE-P/MCH

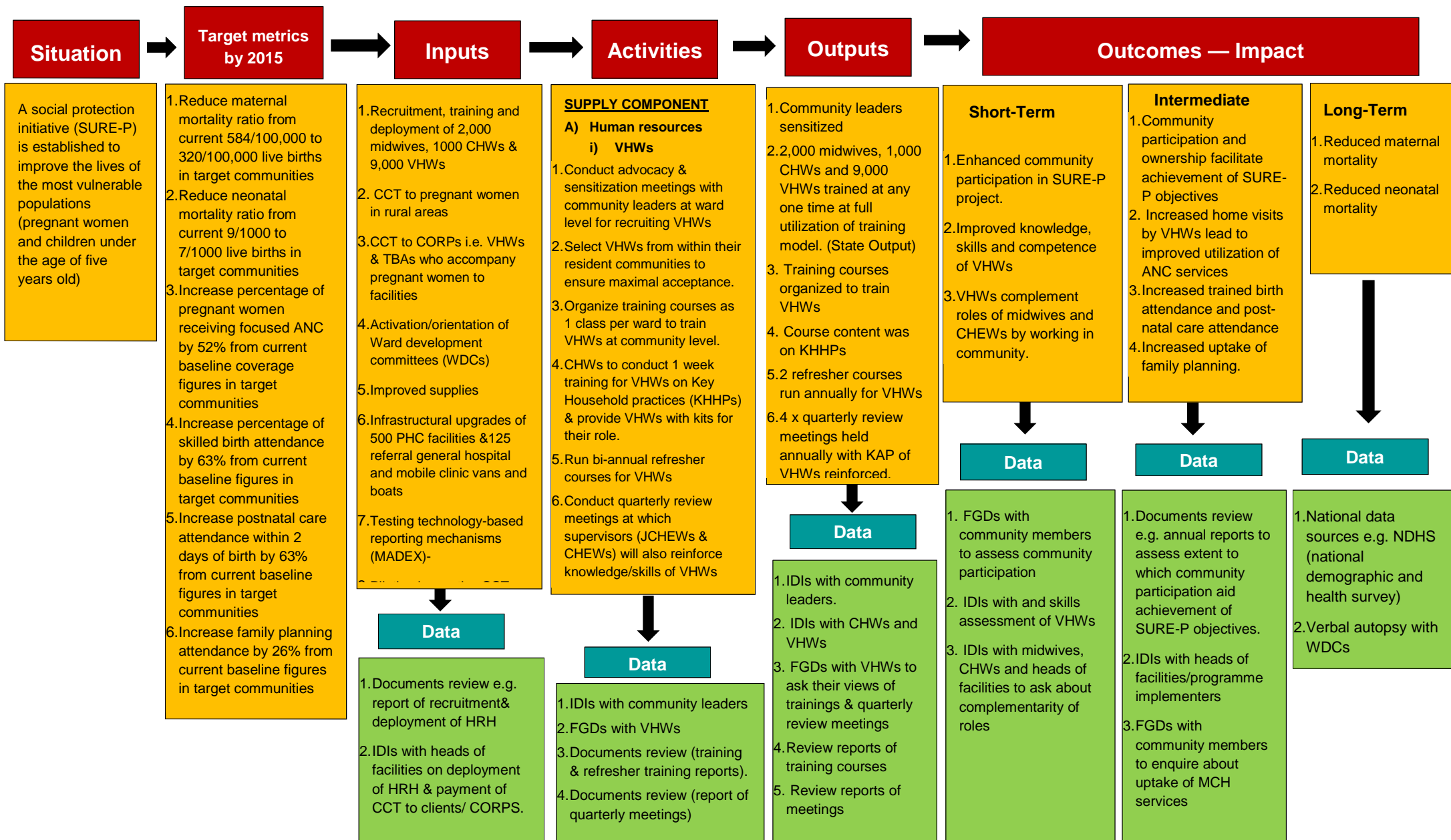

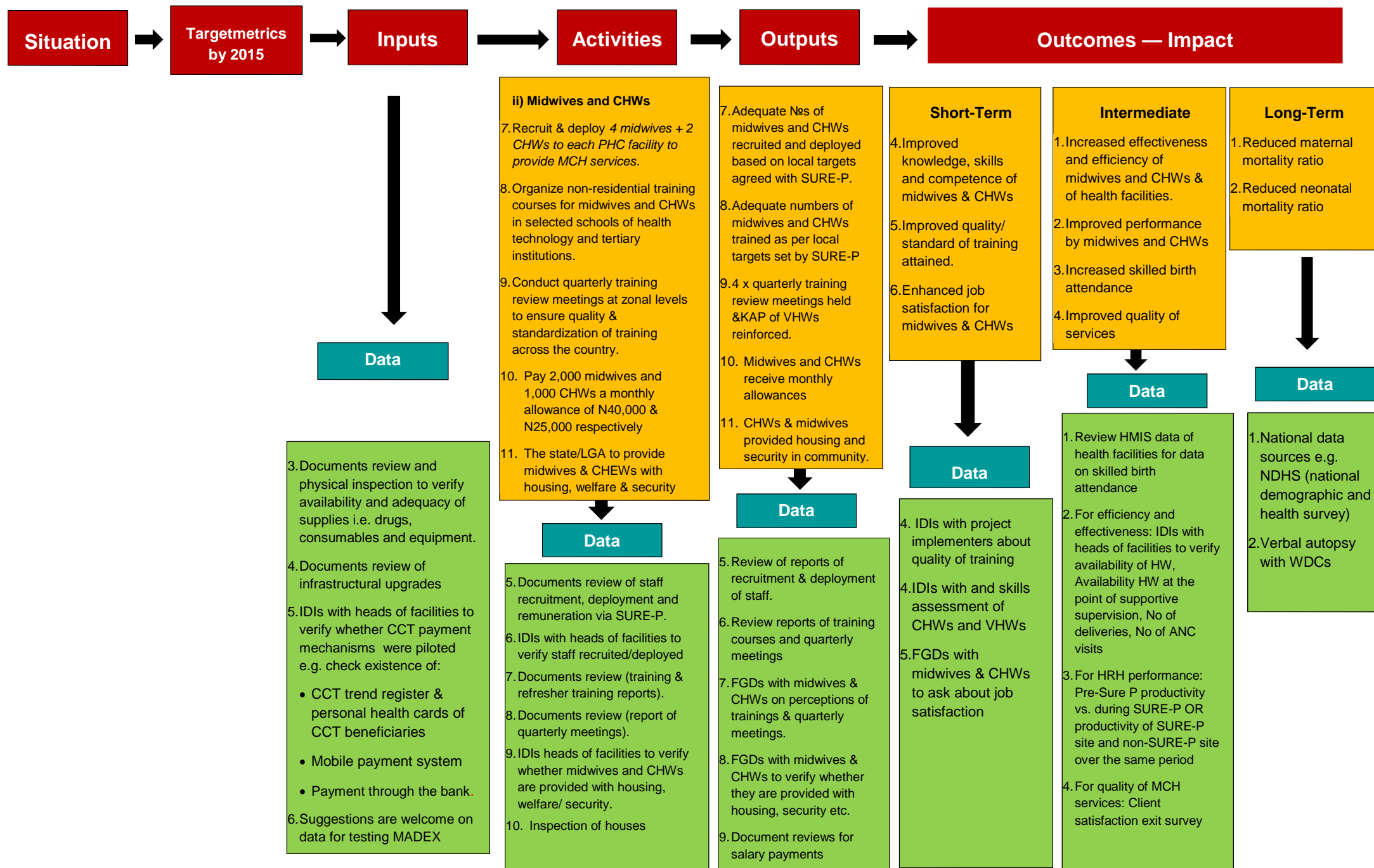

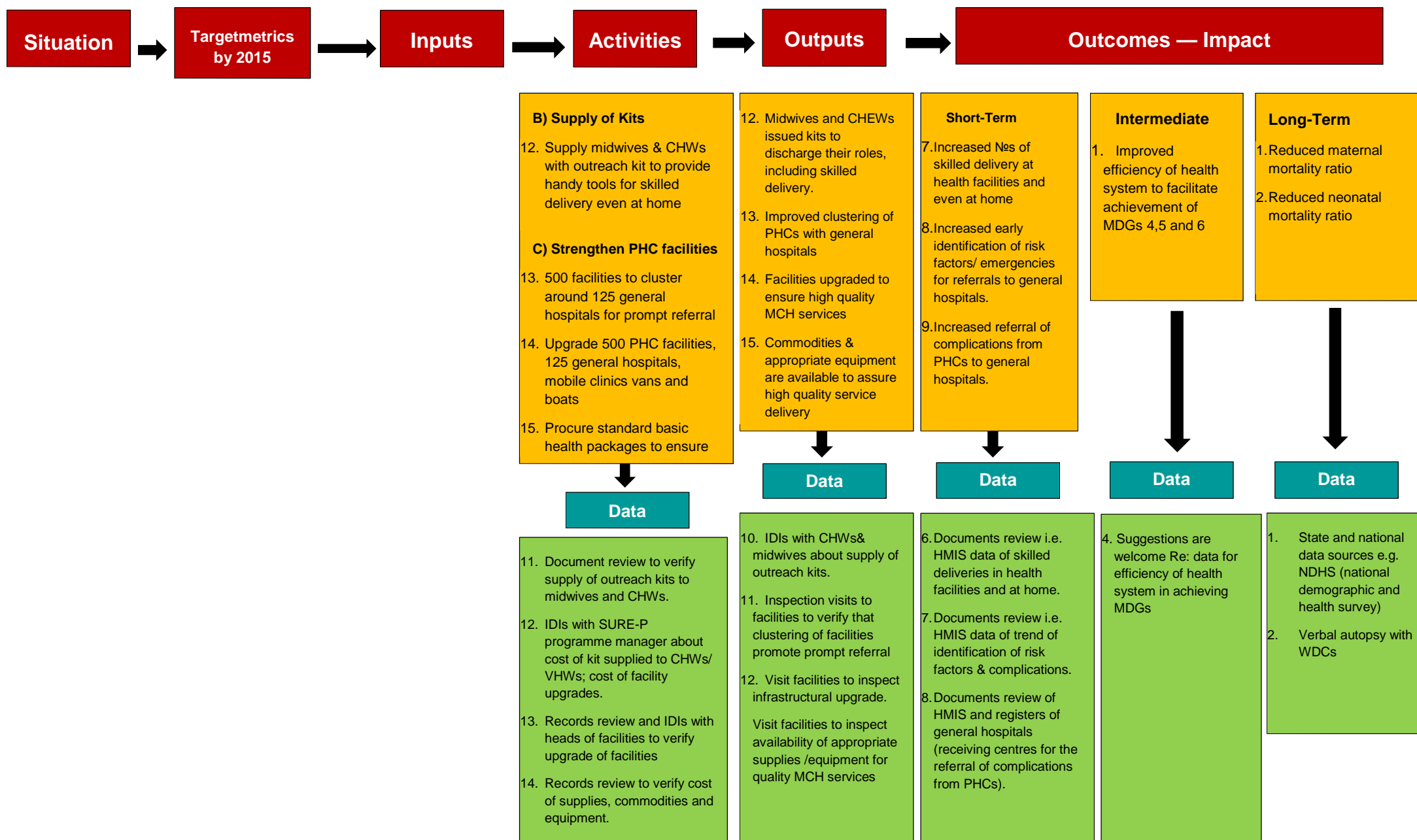

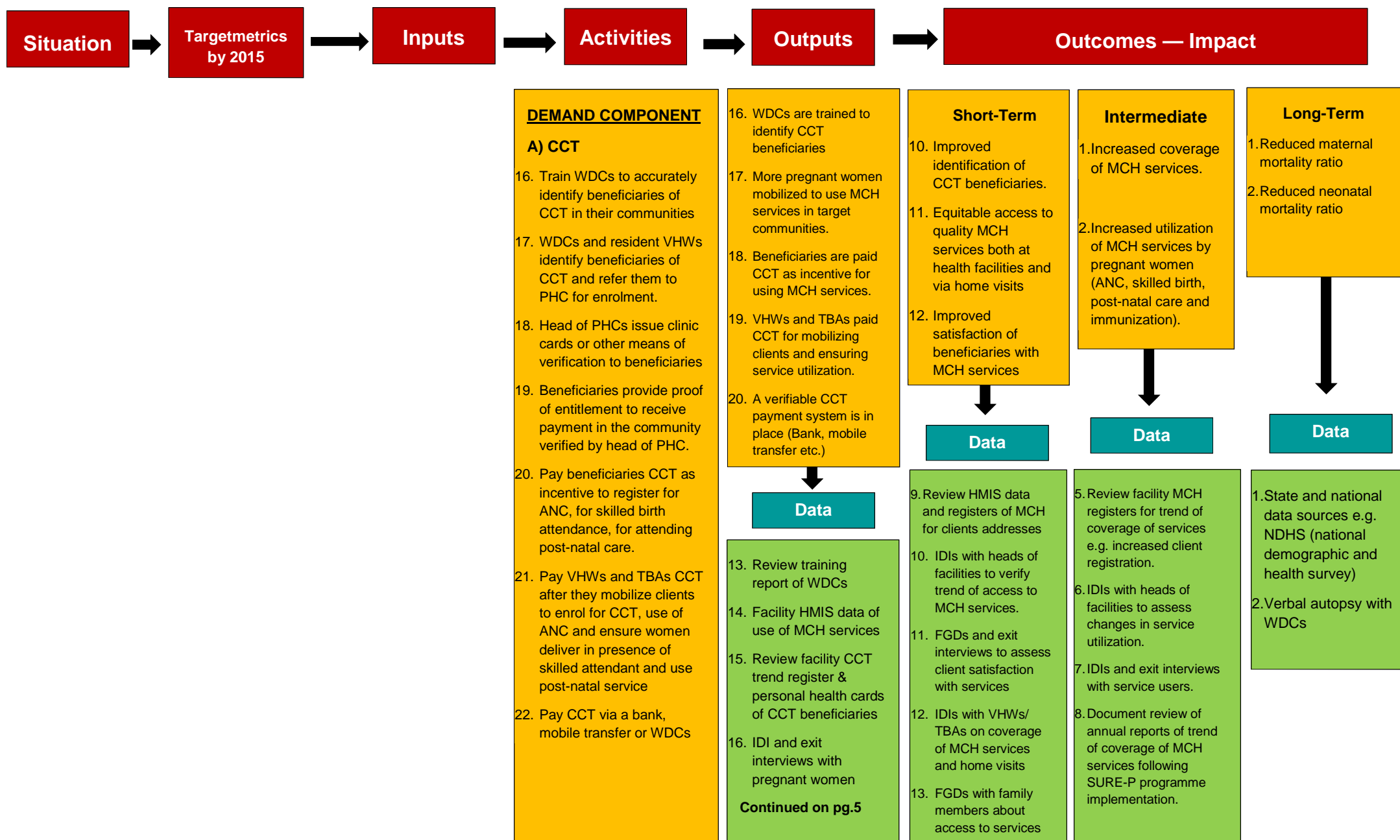

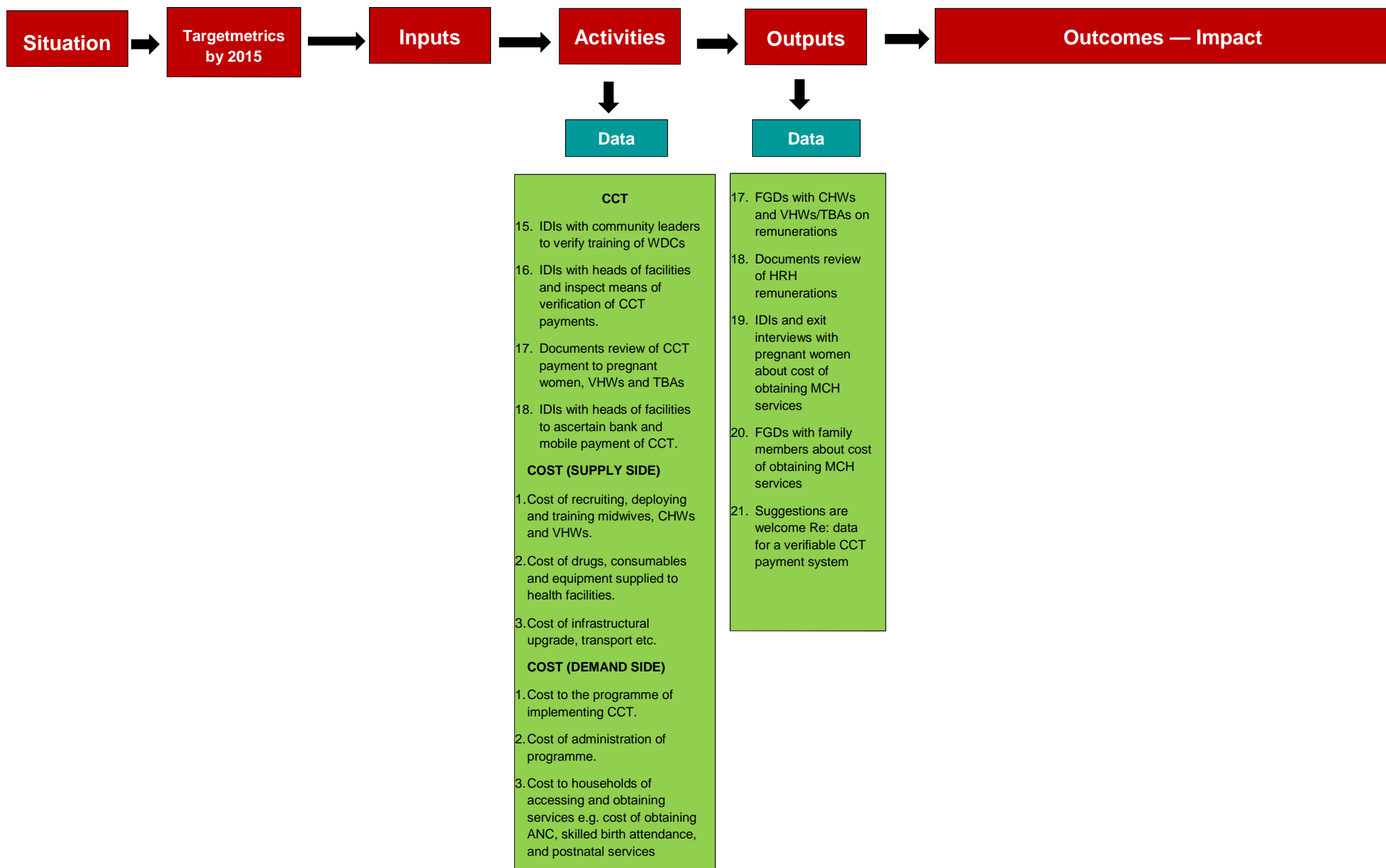

Supplement: Supplementary file 5 — Pre-implementation Logic Map for SURE-P/MCH. (PDF 110 kb) [file 13012_2016_443_MOESM5_ESM.pdf]
