# Supplementary material for: Study protocol: realist evaluation of effectiveness and sustainability of a community health workers programme in improving maternal and child health in Nigeria
Source: Implement Sci. 2016 Jun 7;11:83. doi: 10.1186/s13012-016-0443-1 (PMC4896007; doi:10.1186/s13012-016-0443-1)
Supplement: Supplementary file 6 — Initial Working Theories. (DOCX 37 kb) [file 13012_2016_443_MOESM6_ESM.docx]

**Additional file 6: Initial working theories (IWTs) for the supply and demand components of the SURE-P/MCH programme**

IWT for the supply component

| **IWT:** If different incentives (e.g. regular payments, training and improved working environment) are available in a timely manner, this will lead to improved and sustained health worker motivation, job satisfaction, performance and improved retention of staff in the context of Anambra State which amongst other issues is characterised by irregular salaries and poorly functioning facilities. | | |
| --- | --- | --- |
| C1 Non-experienced staff experience  C2 Status and skill mix of MCH staff (CHWs, CHEWs, midwives)  C3 Irregular salaries  C4 Poorly functioning facilities  C5 Strained working relationships between CHEWs and nurses following policy change in PHC facility management  C6 New Government policy on social protection of vulnerable populations implemented as a pilot | M1 Availability health workers and skill mix of MCH staff ensured  M2 Continuous training of staff  M3 Supportive supervision of staff  M4 Collegial working environment  M5 Regular payment are instituted  M6 availability of equipment supplies and infrastructure  M7 Availability of SURE-P regulatory oversight | O1 Altruism and increased social responsibility  O2 Increased staff motivation  O3 Increased satisfaction  O4 Improved staff performance  O5 Increased staff retention  O6 Improved quality of care delivered by facility  O7 Increased utilization of ANC by women;  O8 Increased skilled birth attendance.  O9 Reduced maternal mortality rate  O10 Reduced infant mortality rate |

IWT for the demand component

| **IWT:** If communities in Anambra State (with poorly-functioning WDCs, irregular payment of incentives and who are unaware of what MCH services are available), are mobilized and financially incentivized in a timely manner, this can lead to improved identification of women, increased coverage and improved utilization of MCH services in a sustainable way. | | |
| --- | --- | --- |
| C1 Community members unaware of MCH services  C2 CHWs are familiar with community context  C3 Poorly functioning WDCs  C4 Irregular payment of incentives  C5 Inequitable geographical access to services | M1 Community members value sensitization messages to help them decide about using MCH services  M2 CHWs build trusting relationships with pregnant women  M4 Collective mobilization of WDCs  M5 Regular payment of incentives to WDCs and CHWs/VHWs  M6 Policymakers appreciate the need to invest in availability of Mama kits to VHWs  M7 SURE-P regulatory oversight | O1 Individual empowerment of community members to demand services  O2 Confidence in MCH services  O3 Positive behaviour change reflected as increased utilization of services  O4 Collective empowerment of WDCs  O5 Improved identification of pregnant women  O6 Reduced maternal mortality rate  O7 Reduced infant mortality rate |

*Explanatory notes:*

Each IWT above is complex and represents a combination of different contexts, mechanisms and outcomes. In other words, more detailed theories can be developed from each of the above IWTs, drawing on different combinations of Cs, Ms and Os. For example, the supply IWT can be broken down into the following, supported by relevant literature:

1. In poorly functioning facilities, if the right material resources are available to motivated senior management who include front-line staff in decision-making, this could lead to increased staff performance and quality of service delivery [1, 2].
2. If different types of incentives (e.g. competence training, regular salaries and supportive supervision), are provided to demoralized health staff in poorly functioning facilities, this can lead to improved staff motivation and performance reflected in improved quality of service [3].
3. In the context of unfavourable working environment, if facility managers create a cordial atmosphere and provide supportive supervision, this can lead to improved staff motivation and job satisfaction [4, 5].
4. In the context of poor social recognition, if the community is mobilized and approval is provided for the roles of CHWs, this can lead to increased credibility and motivation of CHWs to perform their responsibilities in the community [6, 7].

The demand-side IWT can be developed into the following more specific tentative theories:

1. In the context of widespread poverty, if financial incentives are available to pregnant women, and VHWs form trusting relationships with the women, these can lead to increased use of incentives for health gain and utilization for services [8, 9].
2. In the context of community mistrust of health workers, if VHWs demonstrate empathy and cultural resonance towards pregnant women, this can lead to increased confidence in health service and positive behaviour change reflected in increased utilization of MCH services and institutional delivery [10, 11].
3. In the context of low level of MCH consciousness; and availability of high quality health services and a capable WDC, if the community is sensitized and mobilized, this can lead to increased patient/community empowerment reflected as self-/community-efficacy and increased use of MCH services [11]

References

1. Kumar S, Dansereau E: **Supply-Side Barriers to Maternity-Care in India: A Facility-Based Analysis.** *PLoS ONE* 2014, **9:**e103927.

2. Kyei-Nimakoh M, Carolan-Olah M, McCann T: **Barriers to obstetric care at health facilities in sub-Saharan Africa - a systematic review protocol.** *Systematic Reviews* 2015, **4:**54.

3. Singh D, Negin J, Otim M, Orach C, Cumming R: **The effect of payment and incentives on motivation and focus of community health workers: five case studies from low- and middle-income countries.** *Human Resources for Health* 2015, **13:**58.

4. Smith S, Agarwal A, Crigler L, Gallo M, Finlay A, Homsi FA, Lanford E, Wiskow C, Wuliji T: **Community Health Volunteer Program Functionality and Performance in Madagascar: A Synthesis of Qualitative and Quantitative Assessments.** Bethesda, MD: USAID Health Care Improvement Project.; 2013.

5. Kok MC, Dieleman M, Taegtmeyer M, Broerse JE, Kane SS, Ormel H, Tijm MM, de Koning KA: **Which intervention design factors influence performance of community health workers in low- and middle-income countries? A systematic review.** *Health Policy and Planning* 2014.

6. Bagonza J, Kibira S, Rutebemberwa E: **Performance of community health workers managing malaria, pneumonia and diarrhoea under the community case management programme in central Uganda: a cross sectional study.** *Malaria Journal* 2014, **13:**367.

7. Strachan D, Kallander K, Nakirunda M, Ndima S, Muiambo A, Hill Z, group tis: **Using theory and formative research to design interventions to improve community health worker motivation, retention and performance in Mozambique and Uganda.** *Human Resources for Health* 2015, **13:**25.

8. Murray S, Hunter B, Bisht R, Ensor T, Bick D: **Effects of demand-side financing on utilisation, experiences and outcomes of maternity care in low- and middle-income countries: a systematic review.** *BMC Pregnancy and Childbirth* 2014, **14:**30.

9. Okoli U, Morris L, Oshin A, Pate M, Aigbe C, Muhammad A: **Conditional cash transfer schemes in Nigeria: potential gains for maternal and child health service uptake in a national pilot programme.** *BMC Pregnancy and Childbirth* 2014, **14:**408.

10. Østergaard LR: **Trust matters: A narrative literature review of the role of trust in health care systems in sub-Saharan Africa.** *Global Public Health* 2015, **10:**1046-1059.

11. van der Kwaak A, Ferris K, van Kats J, Dieleman M: **Performances of sexuality counselling: A framework for provider–client encounters.** *Patient Education and Counseling* 2010, **81:**338-342.
